# The gut microbiota in Graves’ disease: mechanistic insights and clinical implications

**DOI:** 10.3389/fcimb.2026.1859658

**Published:** 2026-09-01

**Authors:** Leyang Li, Boxian Pang, Ze Wang, Di Zhao, Junping Wei, Qiuhong Wang

**Affiliations:** 1 Department of Endocrinology, Guang’anmen Hospital, China Academy of Chinese Medical Sciences, Beijing, China; 2 Third Affiliated Hospital of Beijing University of Chinese Medicine, Beijing, China

**Keywords:** Graves’ disease, gut microbial metabolites, gut microbiota, gut–thyroid axis, thyroid autoimmunity

## Abstract

Graves’ disease (GD) is an autoimmune form of hyperthyroidism characterized by loss of immune tolerance to the thyrotropin receptor and sustained thyroid hormone excess. Interest in the gut–thyroid axis has expanded rapidly, placing the gut microbiota within current models of GD pathophysiology. This review summarizes current evidence on gut microbial alterations in GD and discusses how these changes may intersect with thyroid autoimmunity. Available studies broadly support disruption of the intestinal microbial ecosystem in GD, although findings for individual taxa and diversity indices vary across cohorts. Proposed links between dysbiosis and disease include altered short-chain fatty acid and bile acid metabolism, impairment of epithelial barrier integrity with translocation of microbial products, shifts in Th17/Treg balance and related immune activation, molecular mimicry, and disturbed handling of micronutrients involved in thyroid hormone synthesis and metabolism. The oral–gut microbial connection has also emerged as a potentially relevant dimension of disease-associated dysbiosis. In parallel, microbiota-directed approaches, including probiotics, prebiotics, synbiotics, dietary modulation, and fecal microbiota transplantation, are being explored as possible adjuncts in GD management. Overall, gut microbial disturbance offers a biologically plausible link between environmental exposure, immune disequilibrium, and thyroid dysfunction in GD; however, stronger mechanistic, longitudinal, and interventional evidence is still required before these findings can be translated into precision clinical practice.

## 1 Introduction

As the primary cause of hyperthyroidism, Graves’ disease (GD) is an autoimmune condition arising from a loss of immunological tolerance to the thyroid-stimulating hormone receptor (TSHR). Autoantibodies bind to and stimulate the TSHR on the surface of thyrocytes, leading to excessive and autonomous production of thyroid hormones (THs). This process results in thyrotoxicosis and a series of clinical manifestations, including tachycardia, hypermetabolism, heat intolerance, and fatigue (Lane et al., 2020). Epidemiologically, in regions with adequate iodine intake, the annual incidence of GD varies between 20 and 30 cases per 100,000 inhabitants, with a marked gender disparity—women are affected 5 to 6 times more frequently than men. Orbital imaging reveals subtle abnormalities in approximately 70% of patients with GD. Clinically apparent Graves’ orbitopathy (GO) is observed in approximately 40% of patients with GD and may present with proptosis, periorbital erythema and edema, orbital pain, diplopia, visual impairment, and corneal involvement (Smith and Hegedüs, 2016; Wiersinga et al., 2025).

The human body harbors trillions of microorganisms that are integral to human health. As an important immune organ, the gut is closely linked to host immune homeostasis. Disruption of the gut microbial balance has been associated with immune dysfunction, which is a well-recognized contributor to autoimmune thyroid diseases, including GD (Wang et al., 2024a). Based on accumulating evidence, the concept of “gut-thyroid axis” has been proposed to describe this close connection. Although associations between gut dysbiosis and thyroid disorders have been widely reported, a definitive causal link has not yet been established. Interpretation of current findings is further complicated by potential confounding factors, including geographic location, ethnicity, and environmental exposures (Virili et al., 2024).

This review summarizes emerging evidence on the interactions within the gut-thyroid axis. We begin by delineating the fundamental pathophysiology of GD and the essential characteristics of the gut microbiota. Subsequently, we discuss the specific compositional alterations and dysbiosis observed in the gut microbiome of GD patients, elucidating the potential underlying mechanisms. Finally, we assess the prospective utility of the gut microbiota as a source of novel diagnostic biomarkers and as a promising therapeutic target for the clinical management of GD.

## 2 Pathogenesis of GD

GD arises from a complex interplay between inherited susceptibility and environmental exposure (Zhang et al., 2024c). Genetic variation may influence immune regulation, thyroid hormone secretion, and apoptosis, and multiple susceptibility loci have been implicated in disease onset (Vargas-Uricoechea, 2023). Among these, rs179247, located in intron 1 of the TSHR gene, has consistently been identified as a locus associated with GD risk (Ploski et al., 2010; Qian et al., 2016; Sun et al., 2019). A recent meta-analysis reported significant associations between rs179247 polymorphism and GD across several genetic models (Zufry and Hariyanto, 2024). In parallel with genetic predisposition, epigenetic regulation—including DNA methylation, histone modification, and non-coding RNAs—has also been implicated in GD by reshaping disease-related gene expression patterns (Vargas-Uricoechea, 2023).

Environmental triggers include infection, nutritional status, endocrine disruptors, iodine intake, selenium deficiency, vitamin D deficiency, smoking, alcohol intake, and psychological stress. In this autoimmune context, antigen presenting cells (APCs), including B cells, macrophages, dendritic cells, recognize TSHR-derived peptides and present them through major histocompatibility complex (MHC) molecules, thereby promoting T-cell activation. In addition, thyroid epithelial cells in GD express MHC class II molecules and CD40, suggesting that they may also participate in local antigen presentation. While the pathogenesis of GD has traditionally been framed within a Th1/Th2 dichotomy, the predominant driver remains a subject of debate. Current evidence points toward a Th2-mediated humoral immune response as the primary mechanism. By stimulating B cells, this response promotes the production of TSHR autoantibodies, which ultimately drive thyrocyte hyperfunction and clinical hyperthyroidism (Chaker et al., 2024; Lanzolla et al., 2024). Recent studies have highlighted the importance of Th17/Treg disequilibrium in the immunopathogenesis of GD. In many patients, this imbalance is characterized by an increased proportion of Th17 cells and a reduced proportion of Treg cells in peripheral blood, suggesting a shift from immune tolerance toward proinflammatory activation. Th17 cells exert pathogenic effects mainly through IL-17A and related cytokines, whereas Treg cells maintain immune homeostasis by suppressing excessive immune responses through cytokine-dependent and cell-contact-dependent mechanisms (Wang et al., 2023b). In GD, IL-17A signaling has been shown to induce the expression of IL-6, CXCL10, and ICAM-1 in thyrocytes, thereby amplifying the local inflammatory milieu and facilitating the recruitment and retention of immune cells. Moreover, activated Th17 cells have been associated with thyroid autoantibody levels, thyroid hormone excess, thyroid enlargement, and the risk of intractable or recurrent disease. However, whether Th17 cells directly promote thyroid follicular-cell proliferation through TSHR-independent pathways remains to be fully elucidated (Shao et al., 2018; Torimoto et al., 2022; Wang et al., 2023d).

## 3 Gut microbiota

### 3.1 Basic characteristics of gut microbiota

The human gut is the habitat of approximately 10 trillion symbionts, including bacteria, archaea, fungi, viruses and phages, with approximately 150–170 bacterial species dominating and performing protective, metabolic and structural functions (Adak and Khan, 2019; Wang et al., 2023c). Microbial communities vary markedly along different regions of the gastrointestinal tract. Alterations in intestinal biogeography have been associated with disease (McCallum and Tropini, 2024). The normal gut barrier consists of several components such as surface mucus, the epithelium and immune defenses that prevent harmful substances in the gut from entering the circulatory system. The gut microbiota is essential for sustaining intestinal barrier integrity. These microbes help preserve epithelial integrity and modulate mucosal immunity, inhibiting pathogen colonization while regulating immune responses through their metabolic byproducts, structural components, and interactions with host cells. On the one hand, epithelial cells form chemical and physical barriers to segregate gut bacteria from immune cells, fostering a mutually beneficial relationship. On the other hand, intestinal immune cells contribute to maintaining a balanced microbial ecosystem, which in turn strengthens epithelial defense mechanisms (Kayama et al., 2020; Martel et al., 2022). In adults, Firmicutes and Bacteroidetes constitute the dominant bacterial phyla in the gut microbiota. Although these phyla are functionally heterogeneous, the Firmicutes/Bacteroidetes (F/B) ratio is often used as a broad, albeit simplified, indicator of gut microbial dysbiosis (Magne et al., 2020; Tsai et al., 2025). The gut ecosystem is not limited to bacteria but also includes fungi, protists, archaea, viruses, and other important non-bacterial members. Although fungi account for only 0.01%–0.1% of the gut microbiota, their relatively larger cell size, greater morphological diversity, and distinct metabolic and immunoregulatory properties suggest that they may exert biological effects disproportionate to their abundance. Recent studies have identified stable fungal enterotypes associated with bacterial enterotypes, highlighting the multi-kingdom structure of the gut ecosystem (Costea et al., 2018; Lai et al., 2023). These non-bacterial members may influence microbial ecology and mucosal immune homeostasis through interactions with bacterial communities and host immune pathways (Ost and Round, 2023; Huang et al., 2024).

### 3.2 Influencing factors of the gut microbiota

In healthy adults, the gut microbial ecosystem maintains a relatively stable state. However, this stability can be perturbed by external and internal factors, including geographic location, dietary habits, smoking, medications and the aging process.

Dietary habits and geographical provenance are recognized as significant factors correlated with variations in microbial composition. Dietary patterns are potent modulators of the gut microbial ecosystem; long-term intake of high-sugar and high-fat diets, along with excessive alcohol consumption, has been shown to adversely alter the microbial community structure and deplete beneficial bacteria and their metabolites, whereas the Mediterranean diet helps foster a beneficial microbial environment (Zmora et al., 2019; Ross et al., 2024). Further studies suggest that geography, urbanization, ethnicity, and dietary habits can also influence the structure of the gut mycobiome, indicating that non-bacterial members may similarly be shaped by lifestyle and environmental factors (Sun et al., 2021; Schaan et al., 2021; Vinogradova et al., 2024).

Cigarette smoking is a crucial clinical risk factor for GD, particularly GO, and has also been reported to alter the gut microbiota and impair intestinal barrier function (Bai et al., 2022; Fan et al., 2025). Medication exposure is another factor to consider in microbiome research; antibiotic-mediated intestinal microbial dysbiosis is associated with various gastrointestinal inflammatory and autoimmune diseases (Fishbein et al., 2023; Taitz et al., 2025).

Recent studies have introduced the novel concept of “gut microbial age”, positioning the gut microbiome as a highly promising novel biomarker for evaluating biological aging trajectories (Wang et al., 2024c). This may intersect with age-related alterations in overall immune and endocrine functions, including thyroid hormone action (van Heemst, 2024), although the underlying mechanisms—particularly the specific role played by the gut microbiota—remain relatively vague.

### 3.3 Functional aspects of the gut microbiota

The gut ecosystem, composed of host tissues, microbial communities, and their metabolic byproducts, exerts systemic regulatory effects on distant organ systems by modulating immune-cell activity, maintaining intestinal barrier integrity, and acting through other incompletely defined pathways, thereby influencing host metabolism, immunity, and related physiological processes (Lin et al., 2025). The gut microbiota modulates host physiology through the synthesis of multiple bioactive molecules, mainly including short-chain fatty acids (SCFAs), bile acid metabolites, and tryptophan derivatives. These microbial metabolites exert multifaceted effects, encompassing nutrient metabolism, energy balance regulation, immune system maturation, immune cell metabolism, and the balance between immunoregulatory and pro-inflammatory cell populations (Wang et al., 2023c). SCFAs originate from the bacterial fermentation of indigestible polysaccharides and dietary fiber and not only serve as primary energy substrates for colonic epithelial cells but also enhance intestinal barrier integrity by upregulating key tight-junction proteins, including claudin-1, claudin-7, and zonula occludens-1. Immunologically, SCFAs exert dual regulatory effects by promoting the clonal expansion and functional maturation of regulatory T cells (Tregs) while suppressing the secretion of inflammatory cytokines (Hays et al., 2024; Mann et al., 2024). The gut serves as the primary reservoir for Gram-negative bacteria, whose outer membranes are rich in lipopolysaccharide (LPS). Under physiological conditions, commensal-derived LPS participates in the maintenance of host immune tolerance. When the epithelial barrier is impaired, LPS can translocate into the bloodstream, leading to excessive production of pro-inflammatory cytokines and ultimately contributing to systemic endotoxemia and chronic low-grade inflammation. LPS leakage and subsequent immune recognition represent the pivotal nexus bridging gut barrier dysfunction to systemic inflammation (Di Lorenzo et al., 2022; Zhang et al., 2024d).

## 4 Gut microbiota changes in GD

Both the thyroid and the gastrointestinal tract share a common embryonic origin, arising from the primitive foregut endoderm. This shared lineage is reflected in their physiological similarities, including active iodine transport via the sodium/iodide symporter (NIS), conserved apical-basal cell polarity, and shared molecular signaling pathways, which collectively establish a robust biological foundation for the gut–thyroid axis (Nowotschin and Hadjantonakis, 2020). Over the past few years, systematic studies in animal models of GD and in patients have revealed alterations in the gut microbiota and explored their potential association with disease.

A recently published meta-analysis demonstrated significant reductions in α-diversity indices, including Chao1, ACE, and Shannon, among patients with GD. It also reported a distinct phylum-level shift characterized by decreased Firmicutes and increased Bacteroidetes, resulting in a reduced F/B ratio. In addition, the relative abundance of Prevotellaceae was significantly increased (Zufry et al., 2024). These findings align with earlier observations reported by Alkader et al. (2023). α-Diversity serves as a composite measure of microbial ecosystem complexity, reflecting both species richness (number of distinct taxa) and evenness (distribution of relative abundances) within a given habitat (Parizadeh and Arrieta, 2023). Further supporting these dysbiosis patterns, Gong et al. identified a dual microbial shift in GD patients featuring depletion of anti-inflammatory commensals alongside enrichment of pro-inflammatory pathobionts (Gong et al., 2021).

Through a comprehensive literature survey, we summarized representative studies on gut microbial changes in GD/GO status, including 22 clinical studies and two animal model studies. The relevant results are shown in **Table 1**. Some studies used subjects diagnosed with autoimmune thyroid disease (AITD), and we extracted only the data on GD/GO from them.

**Table 1:** Studies on gut microbiota alterations in Graves’ disease.

First author/publication year	Country/design/sample size	Microbial sequencing methods	Community-level findings	Key differential taxa
Qianshi Zhang/ 2023 (Zhang et al., 2023)	China/cross-sectional; severity-stratified/GO 62 (mild 20, moderate-to-severe 25, sight-threatening 17), HC 18	16S rRNA gene V3–V4 (Illumina HiSeq)	Sobs: NSD; Chao1: NSD; ACE: NSD; Shannon: NSD; Simpson: NSD; β-diversity: SD; F/B: NSD	↑ *Klebsiella pneumoniae* (severity-associated)
Yuanyuan Deng/ 2023 (Deng et al., 2023)	China/prospective observational/newly diagnosed untreated GD 65, HC 33	16S rRNA gene V3–V4 (Illumina MiSeq)	Observed ASVs: ↓; Faith’s PD: ↓; Shannon: ↓; Pielou’s evenness: NSD; β-diversity: SD; F/B: NSD	↑ *Streptococcus*, *Veillonella*, *Erysipelatoclostridium*; ↓ *Adlercreutzia*, *Roseburia*
Hong Zhao/ 2022 (Zhao et al., 2022)	China/cross-sectional; untreated GD 27, HC 16	16S rRNA gene V3–V4; Illumina MiSeq PE300	Sobs: NSD; Simpson: NSD; Shannon: NSD; β-diversity: SD; F/B: NM	↑ Erysipelotrichia, Cyanobacteria, *Ruminococcus*_2; ↓ Bacillaceae, *Megamonas*
T.-T. Shi/ 2019 (Shi et al., 2019)	China/cross-sectional/ severe and active GO 33 (31 on ATDs), HC 32	16S rRNA gene V4; Ion S5 XL	Simpson: ↓; Shannon: ↓; Chao1: NSD; ACE: NSD; β-diversity: SD; F/B: NM	↑ Bacteroidetes, *Prevotella*; ↓ Firmicutes, *Blautia*, *Anaerostipes*
T.-T. Shi/ 2021 (Shi et al., 2021)	China/cross-sectional/euthyroid GD 30, GO 33, HC 32; hyperthyroid patients received ATDs	16S rRNA gene V4 (515F/806R)	Shannon: ↓ in GD and GO vs HC; Simpson: NM; Chao1: NSD; ACE: NSD; β-diversity: SD for HC–GD, HC–GO, and GD–GO; F/B: NM	GD/GO vs HC: ↑ *Prevotella copri*; GO vs GD: ↑ *Subdoligranulum*, *Bilophila*; ↓ Deinococcus-Thermus, Chloroflexi
Qiyun Zhu/2021 (Zhu et al., 2021)	China/cross-sectional/mild GD I 64, severe GD II 36, HC 62; treatment status NM	Shotgun metagenomic sequencing; Illumina HiSeq 2500 (PE100)	Shannon: GD II ↓ vs HC and GD I; GD I vs HC NM; β-diversity: GD I vs HC NSD; GD II vs HC/GD I SD; F/B: NM	GD II vs HC: ↑ *Eggerthella lenta*, *Streptococcus parasanguinis*; ↓ *Faecalibacterium prausnitzii*, *Bifidobacterium adolescentis*, *Akkermansia muciniphila*
Isabel Cornejo-Pareja/ 2020 (Cornejo-Pareja et al., 2020)	Spain/cross-sectional pilot study/ATD-treated euthyroid GD 9, HC 11	16S rRNA gene V2/V4/V8 and V3/V6-V7/V9; Ion Torrent S5	Pielou’s evenness: ↓; Shannon: NSD; β-diversity: SD F/B: NM	GD vs HC: ↑ Fusobacteriaceae, *Fusobacterium*, *Sutterella*; ↓ *Faecalibacterium*
Hui-xian Yan/2020 (Yan et al., 2020)	China/prospectively recruited cross-sectional study/untreated GD 39, HC 17	16S rRNA gene sequencing; Illumina HiSeq 2500	Sobs: NSD; Chao1: NSD; Shannon: ↓; Simpson: NSD; β-diversity: SD; F/B: NM	↑ *Prevotella*, *Megamonas*, *Veillonella*; ↓ *Ruminococcus*, *Alistipes*
Xinhuan Su/2020 (Su et al., 2020)	China/cross-sectional + mouse FMT experiments/initially untreated GD 58, HC 63 (16S subset: GD 38, HC 37); female BALB/c Ad-TSHR289 model receiving HC- or GD-derived FMT	16S rRNA V1–V2; Illumina HiSeq 2500	ACE ↓; Chao1 ↓; Shannon ↓; Simpson ↓; β diversity: SD; F/B ↓	↑ *Prevotella*, *Streptococcus*; ↓ *Bacteroides*, *Alistipes*, *Bacteroides fragilis*
Yalei Liu/ 2024 (Liu et al., 2024c)	China/cross-sectional + *in vitro* PBMC experiment/untreated newly diagnosed or relapsed GD 52, HC 45 (flow cytometry: GD 33, HC 32; *in vitro*: GD 4, HC 4)	16S rRNA V3–V4; Illumina MiSeq	Shannon: NSD; observed ASVs: NSD; Pielou: NSD; β diversity: SD F/B: NM	↑ *Streptococcus*, *Enterococcus*; ↓ *Bacteroides*, *Dialister*, *Coprococcus*
Xiwen Geng/ 2024 (Geng et al., 2024)	China/cross-sectional three-group study/untreated active GD 20, medication-treated recovered GD 19, HC 20	16S rRNA V5–V7; fungal ITS1; protistan 18S rRNA V4; Illumina MiSeq	Bacteria: Sobs: NSD; Shannon: NSD; β diversity: NSD. Fungi: Sobs: NSD; Shannon: NSD; β diversity: SD. Protists: Sobs: NSD; Shannon: NSD; β diversity: NSD. F/B: NM	Active GD vs recovered GD/HC: ↑ Lachnospiraceae, Eggerthellaceae. Active/recovered GD vs HC: ↑ Saccharomycetales incertae sedis; ↓ Piptocephalidaceae, Spizellomycetaceae
Filippo Biscarini /2023 (Biscarini et al., 2023)	United Kingdom, Italy, Belgium, and Germany/multicenter observational study with cross-sectional and longitudinal components/GD 59, GO 46 (mild 36, moderate-severe 10), HC 41; untreated 35, ATD-treated 70; longitudinal subset 20	16S rRNA V1–V2 plus Bifidobacteria-targeted regions; Illumina MiSeq	GD/GO vs HC: observed OTUs: NM; Chao1: NM; Shannon: NM; β diversity: NSD; F/B: ↑ in GD vs HC and combined GD+GO vs HC.	GD/GO vs HC: ↑ Actinobacteria, *Collinsella*, *Fusicatenibacter*; ↓ Bacteroidetes, *Bacteroides*
Mengxue Yang/ 2019 (Yang et al., 2019)	China/cross-sectional/newly diagnosed untreated GD 15, HC 15	16S rRNA gene amplicon sequencing, variable region NM; Illumina MiSeq, 2 × 300-bp paired-end	ACE: NSD; Chao1: NSD; Shannon: NSD; Simpson: NSD; β-diversity: NM; F/B: ↑	↑ *Oribacterium*, *Mogibacterium*, *Lactobacillus*, *Aggregatibacter*
Shih-Cheng Chang/ 2021 (Chang et al., 2021)	China/cross-sectional; previously diagnosed GD 55, age-, sex- and BMI-matched HC 48; all GD patients initially treated with antithyroid drugs, treatment at sampling NM.	16S rRNA V3–V4; Illumina MiSeq, 2 × 300-bp paired-end	ACE: NSD; Chao1: NSD; Shannon: NSD; Simpson: NSD; β diversity: SD F/B: NM	↑ *Prevotella*_9, *Collinsella*, *Actinomyces odontolyticus*; ↓ *Faecalibacterium*, *Lachnospira*
Wen Jiang/ 2021 (Jiang et al., 2021)	China/cross-sectional/untreated GD 45, age- and sex-matched HC 59	16S rRNA V3–V4; Illumina MiSeq PE300	Sobs: ↓; Chao1: ↓; ACE: ↓; Shannon: ↓; Simpson: ↑; β diversity: SD F/B: NM	↑ Bacteroidetes, *Bacteroides*, *Lactobacillus*; ↓ Firmicutes, *Blautia*
Giulia Masetti/ 2018 (Masetti et al., 2018)	UK and Germany/experimental GD/GO model; female BALB/cOlaHsd mice/Center 1: TSHR 5; Center 2: TSHR 10, βgal 8, untreated 6	16S rRNA V1–V2; Illumina MiSeq paired-end; traditional microbial culture	ACE: ↓; Chao1: NSD; Shannon: NSD, β diversity: SD among TSHR, βgal and untreated groups Longitudinal samples: Chao1: ↓ vs βgal overall and at T4^△^; Shannon: ↓ vs βgal at T1^△^; F/B: ↓ vs βgal at T1, but overall immunization effect NSD	↑ Firmicutes, *Lactobacillus*, *Fusibacter*; ↓ Bacteroidetes, *Bacteroides*
J. Chen/ 2021 (Chen et al., 2021)	China/cross-sectional and longitudinal/untreated GD 15, HC 14; 13 GD patients reassessed after 3–5 months of methimazole treatment	16S rDNA V3–V4; Illumina MiSeq paired-end sequencing	Observed OTUs ↓; Shannon ↓; Simpson ↓; Chao1 NSD; β diversity: NM; F/B: NSD	↑ *Lactobacillus*, *Veillonella*; ↓ Synergistetes, *Phascolarctobacterium*, *Coprococcus*
Hanaa T. El-Zawawy/2021 (El Zawawy et al., 2021)	Egypt/cross-sectional/GD 13, HC 30	Quantitative SYBR Green real-time PCR targeting 16S rRNA of selected bacterial taxa	Shannon: NSD; β diversity: NSD; F/B: ↓	↑ Bacteroidetes, *Prevotella*; ↓ Firmicutes
Wen Jiang/ 2023 (Jiang et al., 2023)	China/cross-sectional/untreated GD 39, HC 48	16S rRNA V3–V4; Illumina MiSeq PE300	Chao1: ↓; Shannon: ↓ β-diversity: SD; F/B: ↓	↑ *Bacteroides*, *Lactobacillus*; ↓ *Blautia*, [*Eubacterium hallii*] group, *Collinsella*
Hafiz Muhammad Ishaq/ 2018 (Ishaq et al., 2018)	China/cross-sectional/GD 27, HC 11	PCR-DGGE (16S V3); targeted qPCR for selected taxa; 16S V3–V4 sequencing in a random subset (10 GD/10 HC; Illumina HiSeq 2500).	Sobs: ↓; Chao1: ↓; ACE: ↓; Shannon: NSD; Simpson: NSD; β-diversity: NM F/B: NM	↑ *Prevotella*_9, *Haemophilus*; ↓ *Alistipes*, *Faecalibacterium*, *Lactobacillus*
Y. Li/ 2023 (Li et al., 2023)	China/mouse model/ BALB/c mice: Ad-TSHR 5, Ad-NC 5, blank 5; microbiome comparison: Ad-TSHR vs Ad-NC	16S rRNA V3–V4; Illumina NovaSeq PE250	Observed OTUs: ↓; Chao1: ↓; ACE: ↓; Shannon: NSD; Simpson: NSD; β diversity: SD; F/B: NM	↑ Bacteroidia; ↓ Clostridia
Chaiho Jeong /2024 (Jeong et al., 2024)	Korea/prospective longitudinal study with external cross-sectional HC/untreated GD 29, paired post-treatment GD 25; external HC 230 for diversity analyses (taxonomy figures report HC 130)	16S rRNA V3–V4; Illumina MiSeq	Chao1: ↓; Shannon: ↓; Simpson: NSD; β diversity: SD; F/B: ↓	↑ Bacteroidota; ↓ *Roseburia*, *Sutterella*, *Parasutterella*, *Akkermansia*
Danyu Wang/2025 (Wang et al., 2025)	China/prospective case-control study with embedded randomized intervention and mouse fecal microbiota transplantation/moderate-to-severe active GO 48, GD without GO 40, HC 36; GO/GD patients receiving methimazole	16S rRNA V3–V4; Illumina MiSeq	Shannon: NSD; Simpson: NSD; ACE: NSD; Chao1: NSD; β diversity: SD for GO vs GD and GO vs HC, but NSD for GD vs HC; F/B: NM	GO-enriched: ↑ *Prevotella*, *Bacteroides*, *Roseburia*, *Phascolarctobacterium*, *Sutterella*
Hong Chao/2025 (Chao et al., 2025)	China/case-control/untreated GD 30, age- and sex-matched HC 30	16S rRNA V3–V4; Illumina HiSeq 2000	Chao1: SD*; observed OTUs: SD*; Shannon: SD*; Simpson: SD*; Pielou evenness: SD*; β diversity: SD; F/B: NM	↑ Bacteroidota, *Prevotella*_9, *Bacteroides plebeius*; ↓ Firmicutes, Proteobacteria

Taxa are reported at different taxonomic levels according to the original studies (e.g., phylum, class, order, family, genus, and species). ↑, increased; ↓, decreased; βgal, β-galactosidase plasmid control; GD, Graves’ disease; GO, Graves’ orbitopathy; HC, healthy controls; F/B, Firmicutes/Bacteroidetes ratio; Sobs, observed species; Chao1, Chao1 richness estimator; ACE, abundance-based coverage estimator; Faith’s PD, Faith’s phylogenetic diversity; NSD, no significant difference; SD, significant difference; NM, not mentioned; ^*^This study presents a contradiction between data and text: Numerical values were higher in GD, whereas the original text described decreased α diversity; ^△^T1 and T4 denote sampling time points 3 weeks after the first immunization and at the end of the 18-week immunization protocol, respectively.

Collectively, these studies suggested notable dysbiosis in the intestinal microbial ecosystem of GD patients; inter-study discrepancies were noted in the compositional changes of particular gut microbial taxa. Among 10 studies examining the F/B ratio, divergent trends were observed: four reported a decreased F/B ratio in GD/GO patients, two noted an increase, and four found no significant difference. These inconsistencies suggest that the F/B ratio should be interpreted cautiously in GD, as it represents only a coarse ecological indicator at the phylum level and may be influenced by differences in study population, disease status, sequencing strategies, and other confounding factors. Eight studies failed to identify meaningful alterations in α-diversity metrics. Among the 21 studies reporting β-diversity results, 16 identified significant differences in all reported comparisons, three yielded comparison- or microbial-kingdom-specific mixed findings, and two found no significant difference.

Several studies have elaborated the significance of microbiota changes from specific perspectives. Zhang et al. (2023) reported that *Klebsiella pneumoniae* abundance was positively associated with GO severity, suggesting that this taxon may be involved in disease-related microbial alterations. Geng et al. (2024) examined gut bacterial and fungal communities in patients with active GD, medication-treated recovered patients, and healthy controls. Compared with bacterial communities, fungal community composition showed more prominent group-level differences and did not fully normalize after clinical recovery. These findings extend the gut–thyroid axis beyond bacteria and highlight the mycobiome as an emerging ecological layer in GD. However, they remain primarily ecological observations and do not yet establish a functional role for fungal alterations in thyroid autoimmunity. On the diagnostic side, several studies have screened specific taxa or taxon combinations thereof as potential biomarkers for GD through random forest algorithms. Regarding therapeutic interventions, Deng et al. (2023) and Jeong et al. (2024) observed a gradual restoration of the gut microbiota toward a healthy state following antithyroid drug (ATD) treatment. A longitudinal analysis by Biscarini et al. (2023) revealed that patients with persistent TRAb positivity exhibited distinct microbial signatures compared to those who achieved TRAb seroconversion after treatment.

Among the human studies summarized in **Table 1**, *Lactobacillus* was enriched in four cohorts (Yang et al., 2019; Jiang et al., 2021; Chen et al., 2021; Jiang et al., 2023) but depleted in Ishaq et al. (2018), while Masetti et al. (2018) also reported enrichment in an experimental GD/GO model. These findings are not necessarily directly comparable. Most of the studies reporting enrichment used 16S rRNA gene sequencing and therefore measured relative abundance, whereas the reduction reported by Ishaq et al. was derived from targeted quantitative PCR measuring bacterial gene-copy numbers per gram of feces. Because relative abundance is compositional, an apparent proportional increase may coexist with an unchanged or reduced absolute bacterial load when the total microbial biomass or the abundance of other taxa changes.

Discordant findings for Firmicutes and the F/B ratio may similarly arise from both biological and methodological heterogeneity. Firmicutes comprises functionally diverse taxa, and the recurrent depletion of several SCFA-associated genera, such as *Blautia*, *Roseburia*, *Faecalibacterium*, and members of the *Eubacterium* group, does not imply a uniform reduction of all Firmicutes members. In addition, a change in the F/B ratio may be driven by the numerator, the denominator, or both; for example, the increased F/B ratio in the European INDIGO cohort (Biscarini et al., 2023) occurred in the context of a marked reduction in Bacteroidetes and should not be interpreted as direct evidence of Firmicutes expansion. Further variation is likely introduced by differences in disease phenotype and stage, including untreated hyperthyroid GD, ATD-treated or euthyroid GD, GO of varying severity, and post-treatment cohorts. These distinctions are relevant because longitudinal studies have shown partial restoration of microbial diversity after ATD treatment (Jeong et al., 2024). Geographic and environmental influences may also be substantial; even genetically similar TSHR-immunized mice subjected to comparable protocols in two independent centers developed different microbial profiles and thyroid phenotypes (Masetti et al., 2018). Finally, the included studies used different 16S variable regions, sequencing platforms, targeted PCR assays, shotgun metagenomics, taxonomic databases, and bioinformatic pipelines, which can yield different taxonomic estimates. Thus, the present evidence supports GD-associated restructuring of the intestinal microbial community; however, standardized longitudinal studies integrating absolute microbial quantification, shotgun metagenomics, and rigorous control of treatment, diet, iodine exposure, and other environmental factors are still needed to resolve these discrepancies.

Several Mendelian randomization studies have provided genetic evidence suggesting potential causal involvement of specific microbial traits in GD. The prevalence of *H. pylori* infection and CagA serology positivity were significantly elevated in GD patients (Figura et al., 2019). Using bidirectional Mendelian randomization, Wang et al. revealed a reciprocal causal relationship between genetically predicted anti-*H. pylori* OMP antibodies and GD, while also indicating that genetic liability to GD was associated with elevated anti-H. pylori UREA levels (Wang et al., 2024b). Utilizing MiBioGen and FinnGen datasets, Liu et al. identified Bacteroidaceae, *Bacteroides*, and *Veillonella* as significant protective factors against GD. Their analysis further revealed potential risk-reduction trends for the *Eubacterium brachy* group and Family XIII AD3011 group, while *Bilophila*, *Collinsella*, and Streptococcaceae were associated with increased risk (Liu et al., 2024a). Similarly, Fang et al. (2024) reported nine protective taxa, including *Akkermansia*, *Bacteroides*, *Victivallis*, and various Verrucomicrobia lineages. Conversely, *Bifidobacterium*, conventionally regarded as a probiotic, was significantly linked to elevated GD risk. Mediation analysis indicated that *Bifidobacterium* may promote GD pathogenesis by modulating immune phenotypes, specifically decreasing CD39^+^ CD4^+^ T cell proportions. Zhang et al.’s findings similarly suggest that *Bifidobacterium* may represent a risk-associated taxon for GD (Zhang et al., 2024b). These findings challenge the conventional dichotomous classification of microbes as strictly ‘beneficial’ or ‘detrimental.’ Instead, microbial functions exhibit context-dependent versatility, wherein their roles are contingent upon disease-specific gut microenvironmental conditions, demonstrating a more nuanced and multifaceted impact in GD.

## 5 Potential mechanisms of the gut microbiota in Graves’ disease

**Figure 1:**
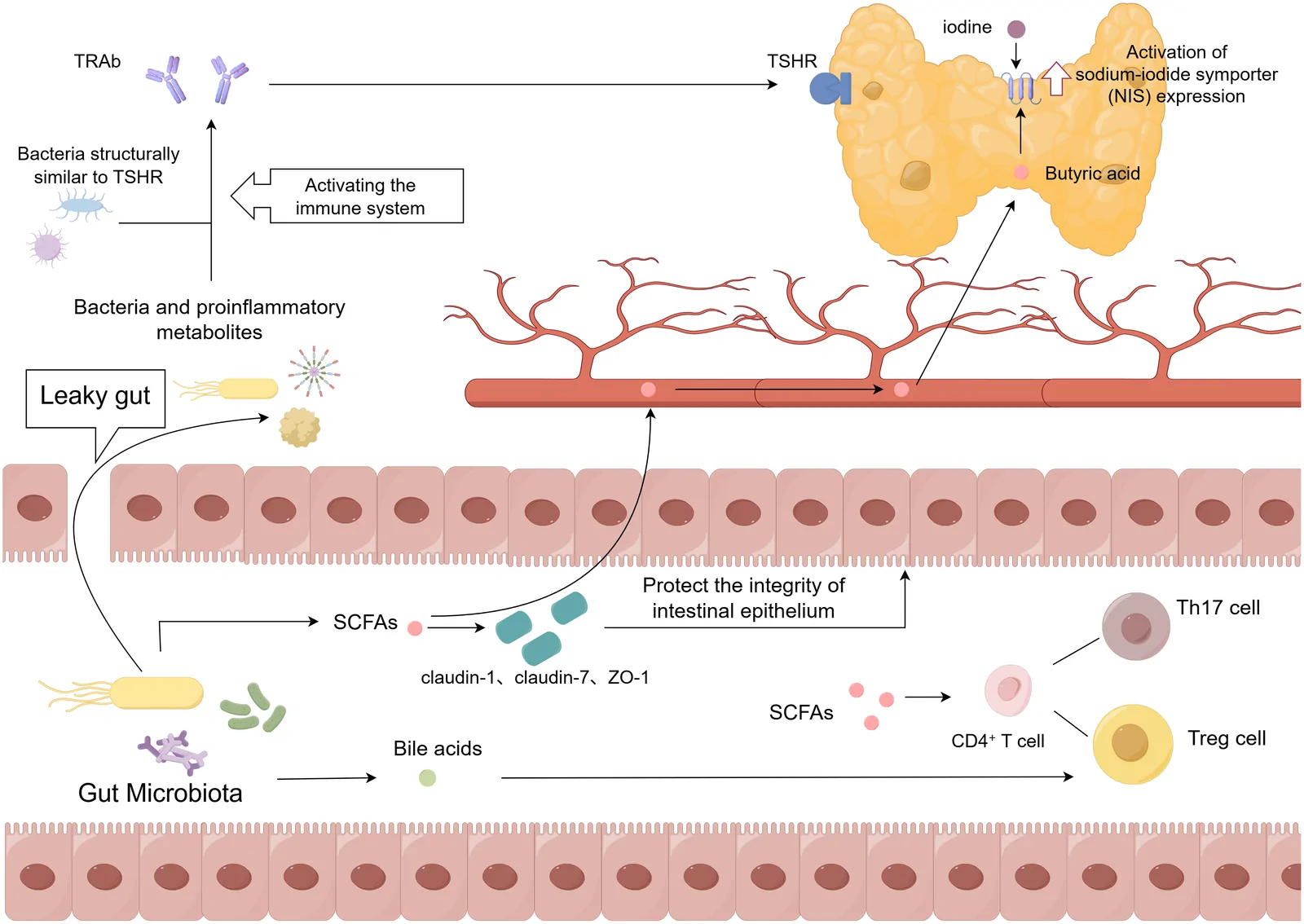
Potential mechanisms by which the gut microbiota influences thyroid health and Graves’ disease. Under physiological conditions, gut microbiota-derived shortchain fatty acids (SCFAs) help preserve intestinal epithelial integrity by upregulating tight junction proteins, including claudin-1, claudin-7, and zonula occludens-1 (ZO-1), and contribute to immune homeostasis by modulating CD4^+^ T-cell differentiation toward a more tolerogenic balance. Microbiota-related metabolites, including butyric acid and bile acids, may also influence thyroid function. Butyrate may promote sodium-iodide symporter (NIS) expression and thereby influence iodine handling. In the setting of Graves’ disease-associated dysbiosis, reduced beneficial microbial metabolites and impaired barrier function (“leaky gut”) may facilitate the translocation of bacteria and pro-inflammatory metabolites into the circulation, promote systemic immune activation, and contribute to an imbalance between Th17 and Treg cells. In parallel, structurally similar microbial antigens may participate in thyroid autoimmunity through molecular mimicry, potentially contributing to thyrotropin receptor antibody (TRAb) generation and TSHR-directed immune responses. Overall, this figure summarizes putative links between gut microbial dysbiosis, epithelial barrier dysfunction, immune imbalance, and thyroid autoimmunity in Graves’ disease. Created by Figdraw.

The gut microbiota may be involved in GD through metabolite-mediated immunomodulation and altered handling of micronutrients essential for thyroid hormone metabolism (**Figure 1**).

### 5.1 Immune pathways

The intestine functions as the principal immune hub in the human body, with its submucosal layer harboring the most concentrated population of immune cells—accounting for 70-80% of the total immune cell count. This regulatory network maintains immune tolerance toward commensal microbiota while promptly identifying pathogenic invaders. It is central to orchestrating intestinal immune defense (Martel et al., 2022; Kulkarni and Newberry, 2025). The functional integrity of the intestinal mucosa and the immune cell profile in mesenteric lymph nodes are shaped by resident microbiota. An overabundance of pathogenic bacteria can amplify pro-inflammatory cytokine release, compromising mucosal barrier function and disrupting immune tolerance, thereby exacerbating systemic inflammation. Through synergistic interactions between bacteria and their metabolites, the balance of key immune cells—including Th17 and Treg cells—is dynamically modulated. This tripartite crosstalk among the microbiota, immunity, and metabolism constitutes a pivotal axis in the gut immunoregulatory mechanisms (Ma et al., 2024).

SCFAs are important microbial metabolites that contribute to intestinal homeostasis by supporting epithelial barrier integrity and regulating mucosal immune responses. Su et al. (2020) found reduced fecal propionate levels in patients with GD and further showed *in vitro* that *Bacteroides fragilis*-derived propionate increased Treg cells while decreasing Th17 cells. These findings suggest that reduced propionate availability may contribute to Treg/Th17 imbalance in GD. Deng et al. (2023) reported significantly lower relative abundances of several putative SCFA-producing taxa. Liu et al. (2024c) identified reduced serum acetate levels and correlations of SCFAs with thyroid autoantibodies, B-cell subsets, and cytokines. They further showed that LPS altered B-cell-subset distribution *in vitro*, whereas a combination of SCFAs attenuated LPS-induced cytokine production. Collectively, these findings suggest that reduced SCFA availability and increased LPS exposure may contribute to Treg/Th17 and B-cell dysregulation in GD; however, a direct causal pathway linking these alterations to TRAb production has not been established.

A bidirectional relationship exists between the intestinal microbial community and bile acid metabolism; specifically, microbial diversity shapes the bile acid profile, whereas bile acid dysmetabolism can, in turn, disrupt the intestinal ecosystem (Pan et al., 2024). After hepatic synthesis, bile acids enter the intestinal lumen in conjugated forms and are subsequently remodeled by the gut microbiota, thereby generating a structurally diverse pool of secondary bile acids, many of which are reabsorbed through enterohepatic circulation. Some anaerobic gut bacteria, especially members of the *Clostridium* group, participate in the generation of major secondary bile acids. Beyond their metabolic functions, bile acids also act as signaling molecules through bile acid receptors, which are expressed in immune cells and intestinal epithelial cells and participate in the regulation of inflammatory responses and barrier integrity. Consistent with this, studies in Fxr^−/−^ and Tgr5^−/−^ mice have shown reduced intestinal barrier integrity and increased bacterial translocation. In addition, specific microbially modified bile acids can shape T-cell differentiation: 3-oxolithocholic acid and isolithocholic acid can directly interact with RORγt and suppress Th17-cell differentiation, whereas other secondary bile acid metabolites, such as isoDCA and isoalloLCA, have been linked to enhanced Treg or peripherally induced Treg responses. Therefore, dysbiosis-driven disruption of bile acid transformation may alter immune homeostasis and intestinal barrier function, providing a plausible route by which the microbiota–bile acid axis contributes to immune dysregulation in GD (Collins et al., 2023; Lee et al., 2024). THs can stimulate the hepatic expression of cholesterol 7α-hydroxylase in the liver. This enzyme is a key enzyme that converts cholesterol into bile acids, thereby promoting the synthesis of bile acids. Bile acids may affect the expression of TH transporters, which are responsible for the transmembrane transport of TH. The changes in their expression and function will affect the absorption efficiency of TH in the intestine and their distribution and metabolism in the body. Therefore, abnormal metabolism of the intestinal microbiota-bile acid axis may affect the metabolism of TH (Sinha and Yen, 2024). Clinical observations from autoimmune cholestatic liver disease further support a potential connection between bile acid dysmetabolism and thyroid disorders. In a multicenter study of 1,554 patients with primary biliary cholangitis (PBC) from 20 international centers, concomitant autoimmune thyroid disease (AITD) was reported in 10.6% of patients, while Graves’ disease accounted for 1.5% of the total cohort (Efe et al., 2021). Jiang et al. (2023) found that bile acids may regulate thyroid function through lipid metabolism and vitamin metabolism. However, few studies have examined how bile acid metabolism may influence GD through the gut-thyroid axis, and more studies integrating microbiome and multi-omics analysis are needed to explore this mechanism in depth.

Various pathogenic factors compromise intestinal epithelial integrity, increasing permeability—a condition termed “leaky gut”. In this state, the gut becomes more permeable to typically restricted substances such as macromolecules, bacteria, and endotoxins, thereby contributing to systemic inflammation in disorders (Camilleri, 2019). One study identified elevated serum LPS and other gut-derived biomarkers in newly diagnosed GD patients, linking heightened intestinal permeability to bacterial translocation and TRAb levels (Zheng et al., 2021). In GO, bacterial product compound translocation due to intestinal barrier dysfunction may further amplify inflammatory cascades within orbital tissues (Fenneman et al., 2023b).

Beyond T-cell polarization, mucosal B-cell responses may provide an additional link between dysbiosis and thyroid autoimmunity. Gut-associated lymphoid tissue continuously samples microbial antigens and generates IgA responses that limit bacterial contact with the epithelium and help shape the microbial community (Bemark et al., 2024). Disruption of this mucosal containment system may increase exposure to microbial products such as LPS. In GD, Liu et al. (2024c) reported altered peripheral B-cell subsets and reduced expression of the inhibitory receptor CD32b. LPS also shifted B-cell subsets *in vitro*, although TRAb production was not examined. Downstream studies of GD have identified increased activated T follicular helper cells and IgD−CD27− double-negative B cells associated with thyroid autoantibodies. CD11c+ B cells also accumulate in the thyroid and can differentiate into TRAb-secreting cells *in vitro* (Torimoto et al., 2022; Cao et al., 2022). These findings support a possible link between mucosal exposure, systemic B-cell activation, and thyroidal autoantibody production, but they do not demonstrate that gut-derived signals activate TSHR-specific B-cell clones.

Molecular mimicry refers to structural or antigenic similarity between exogenous antigens and host self-antigens, which may trigger cross-reactive T-cell or B-cell responses in genetically susceptible individuals, and is regarded as one of the potential mechanisms linking infection to autoimmunity (Rojas et al., 2018). Recent studies on the gut microbiota in extra-intestinal autoimmune diseases have further supported the biological plausibility of molecular mimicry, showing that microbiota-derived peptides can activate autoreactive lymphocytes (Miyauchi et al., 2023; Ma et al., 2025). In GD, infection with *Yersinia* species has long been proposed as a possible contributing factor. A meta-analysis showed that *Yersinia enterocolitica* positivity was significantly associated with autoimmune thyroid disease overall, with a stronger association observed in the GD subgroup (OR = 6.12, 95% CI 3.71–10.10), suggesting a possible role in GD pathogenesis; nevertheless, such evidence remains mainly epidemiological and is insufficient on its own to establish causality. At the mechanistic level, previous studies have suggested that *Y. enterocolitica* infection may induce TSHR-cross-reactive antibodies and T cells (Zangiabadian et al., 2021). An *in vitro* and in silico study demonstrated serological cross-reactivity between OmpF porin from *Yersinia pseudotuberculosis* and human TSHR: monoclonal antibodies against hTSHR were shown to recognize YpOmpF, and molecular modeling further suggested that, despite low overall primary-sequence homology, similar antigenic determinants may arise from local conformational resemblance (Portnyagina et al., 2018). Taken together, these findings support molecular mimicry as an attractive hypothesis linking microbial antigens to thyroid autoimmunity. However, direct evidence linking gut commensal-derived peptides to TSHR-specific T- or B-cell activation in GD is still lacking, and most current support comes from infection-related studies and serological cross-reactivity. Longitudinal studies around disease onset, together with antigen-specific B- and T-cell profiling, are needed to clarify whether such cross-reactive responses precede GD, accompany disease activity, or represent secondary immune phenomena.

### 5.2 Regulation of key micronutrient metabolism

THs serve as master regulators of systemic metabolism and energy homeostasis, exerting regulatory effects on nearly all bodily tissues, including the intestine (Sinha et al., 2025). Following hepatic metabolism, these hormones enter the intestinal lumen via biliary excretion, where they modulate epithelial cell proliferation and differentiation. This process is bidirectionally regulated by the gut microbiota, which facilitate the deconjugation of TH metabolites, enabling triiodothyronine reabsorption through enterohepatic recirculation (Fenneman et al., 2023a). Gut microbial homeostasis influences the bioavailability of essential micronutrients - including iodine, selenium, and iron - all of which serve as critical cofactors for TH synthesis.

Iodine status is a key determinant of the risk of thyroid disease, wherein both insufficient and excessive levels can disrupt normal thyroid function. Excessive iodine may trigger thyroiditis, while iodine deficiency impairs TH synthesis. Variations in iodine intake across different periods and regions have contributed to global fluctuations in thyroid disease prevalence (Taylor et al., 2018). Among adult GD patients following ATD treatment, adequate iodine intake correlates with reduced disease recurrence, enhanced treatment efficacy, and improved control of thyrotoxicosis (Xie et al., 2023). Iodine circulation in TH synthesis includes a series of transport, oxidation, organification and coupling within thyroid follicular cells (Sohn et al., 2024). The transport of active iodide is facilitated by the NIS, a pivotal mediator in TH biosynthesis. In an NHANES-based exposome-wide analysis, NIS inhibitors, including perchlorate and thiocyanate, were associated with altered TH levels across age groups, particularly under chemical co-exposure scenarios (Lee et al., 2025). A recent meta-analysis revealed that exposure to three predominant NIS inhibitors (perchlorate, nitrate, and thiocyanate) was positively associated with TSH levels in adults (Jang et al., 2025). These divergent outcomes likely stem from physiological heterogeneity across studied cohorts and methodological variations in exposure assessment protocols. Butyrate can suppress histone deacetylase activity to upregulate NIS expression in thyroid tissue (Puppin et al., 2005). The LPS signaling axis is a canonical driver of NF-κB activation, which critically modulates NIS transcription. Signals derived from specific ligand-receptor interactions synergize with active NF-κB dimers, ultimately dictating the differential expression profiles of NIS in response to diverse stimuli (Faria et al., 2020).

Iron is required for growth and metabolism of almost all bacterial species, and iron levels significantly affect intestinal microbial composition (Rosell-Diaz et al., 2023). Moderate iron supplementation can stimulate the proliferation of beneficial bacteria, including *Bifidobacterium*, *Akkermansia*, and members of the family Ruminococcaceae, which are important for maintaining intestinal barrier integrity. Conversely, insufficient iron availability diminishes both the quantity and variety of these health-promoting gut microbes (Liu et al., 2024b). Clinical evidence suggests hyperthyroidism significantly increases susceptibility to iron-deficiency anemia (Wopereis et al., 2018; van Vliet et al., 2022). Iron is the core atom of the active site of thyroid peroxidase, a pivotal enzyme involved in TH biosynthesis that mediates iodide ion oxidation during hormone synthesis. Different meta-analyses have yielded conflicting conclusions regarding how iron deficiency influences thyroid physiology, therefore, the relationship between iron homeostasis and GD remains to be fully elucidated (Garofalo et al., 2023; Parsaei et al., 2025).

As an essential trace element, selenium exerts its antioxidant effects through selenoproteins, protecting thyroid cells from oxidative damage. Among human tissues, the thyroid gland maintains the highest physiological selenium concentration in adults. Notably, GD patients exhibit lower selenium levels than healthy controls (Wang et al., 2023a). Selenium upregulates NIS gene transcription, enhances its iodide transport capacity, and counteracts iodine-induced NIS suppression (Oglio et al., 2024). Selenium has a protective effect on the intestinal barrier; selenium compounds may exert a positive impact on intestinal health by promoting the synthesis of selenium nanoparticles (SeNPs) in gut microbiota, especially when the host harbors a high abundance of lactic acid bacteria (Liang et al., 2024). *Lactobacillus* can promote tissue uptake of selenium (Martinez et al., 2020). Researchers found that supplementation with SeNPs-enriched *Lactobacillus casei* can correct phylum-level dysbiosis, increase SCFA levels, and effectively ameliorate intestinal barrier dysfunction by enhancing antioxidant capacity and inhibiting endoplasmic reticulum stress (Song et al., 2022). However, GD-specific evidence is still needed.

### 5.3 Mutual influence between the oral and gut microbiota

Emerging evidence suggests that thyroid functional status may be associated with alterations in the oral microbiota (Dong et al., 2021; Zheng et al., 2023). Located at the proximal end of the digestive tract, the oral cavity harbors a mosaic of site-specific microbial communities shaped by marked differences in surface characteristics, oxygen availability, nutrient exposure, and host defense factors. These communities are not confined to the local oral environment, as oral microorganisms and their products can interact with distal organs through enteral passage, hematogenous dissemination, and immune-mediated pathways. Accordingly, oral dysbiosis is increasingly viewed as a potential upstream driver of downstream intestinal ecological disturbance, particularly when colonization resistance or barrier integrity is compromised (Baker et al., 2024; Kunath et al., 2024; Yamazaki and Kamada, 2024; Xu et al., 2025). The intricate crosstalk between the oral and gut microbiota and their collective interplay with the host has positioned the oral–gut axis as a pivotal frontier in microbiota research. An overabundance of pathogenic oral bacteria may translocate to the gut, disrupting microbial homeostasis and precipitating intestinal inflammation. Among the studies reviewed in **Table 1**, nine reported a significant increase in fecal abundance of *Prevotella* taxa in patients with GD, making enrichment of this genus one of the more recurrent taxonomic signals across otherwise heterogeneous cohorts. This finding is potentially relevant to oral–gut interactions because the oral cavity represents the site of most concentrated phylogenetic diversity of *Prevotella*, and fecal detection of oral-derived *Prevotella* strains suggests a direct oral–gut transmission route. Under pathological conditions, this genus may exacerbate tissue inflammation by driving Th17 cell-mediated immune responses, frequently associated with periodontitis (Schmidt et al., 2019; Tett et al., 2021). Nevertheless, these observations do not establish oral-to-gut transmission in GD. None of the available GD studies combined paired oral and fecal sampling with strain-resolved analysis. *Prevotella* is a phylogenetically and functionally heterogeneous genus, and its effects on host immunity appear to vary according to species, strain, microbial context, and host condition (Tett et al., 2021). Thus, genus-level enrichment should not be interpreted as evidence of either a uniform pro-inflammatory effect or an oral origin. Experimental colitis models provide proof-of-concept evidence that oral pathobionts such as *P. gingivalis* can alter gut microbial ecology and Th17/Treg balance; however, this mechanism has not been investigated in GD (Jia et al., 2024). *Actinomyces odontolyticus*, a dental-associated anaerobe also detected in the gastrointestinal tract, has been shown to induce reactive oxygen species accumulation, mitochondrial dysfunction, NF-κB pathway activation, and direct damage to colonic epithelial cells in colorectal cancer (Miyakawa et al., 2024). Chang et al. (2021) reported an increased fecal abundance of *Actinomyces odontolyticus* in patients with GD, with its abundance positively correlated with TPOAb and FT4 but negatively correlated with TSH; complementing this taxonomic observation, Liu et al. (2024c) found that other GD-associated microbial taxa and SCFAs were correlated with thyroid autoantibodies, inflammatory mediators, and B-cell subsets, further linking gut dysbiosis to endocrine and immune disturbances in GD. Taken together, current evidence provides biological plausibility. Paired oral and fecal metagenomic sequencing, together with longitudinal strain tracking and immune phenotyping, will be required to determine whether oral-derived microorganisms contribute to intestinal dysbiosis or immune dysregulation in GD.

## 6 Treatment and application prospects related to the gut microbiota

**Figure 2:**
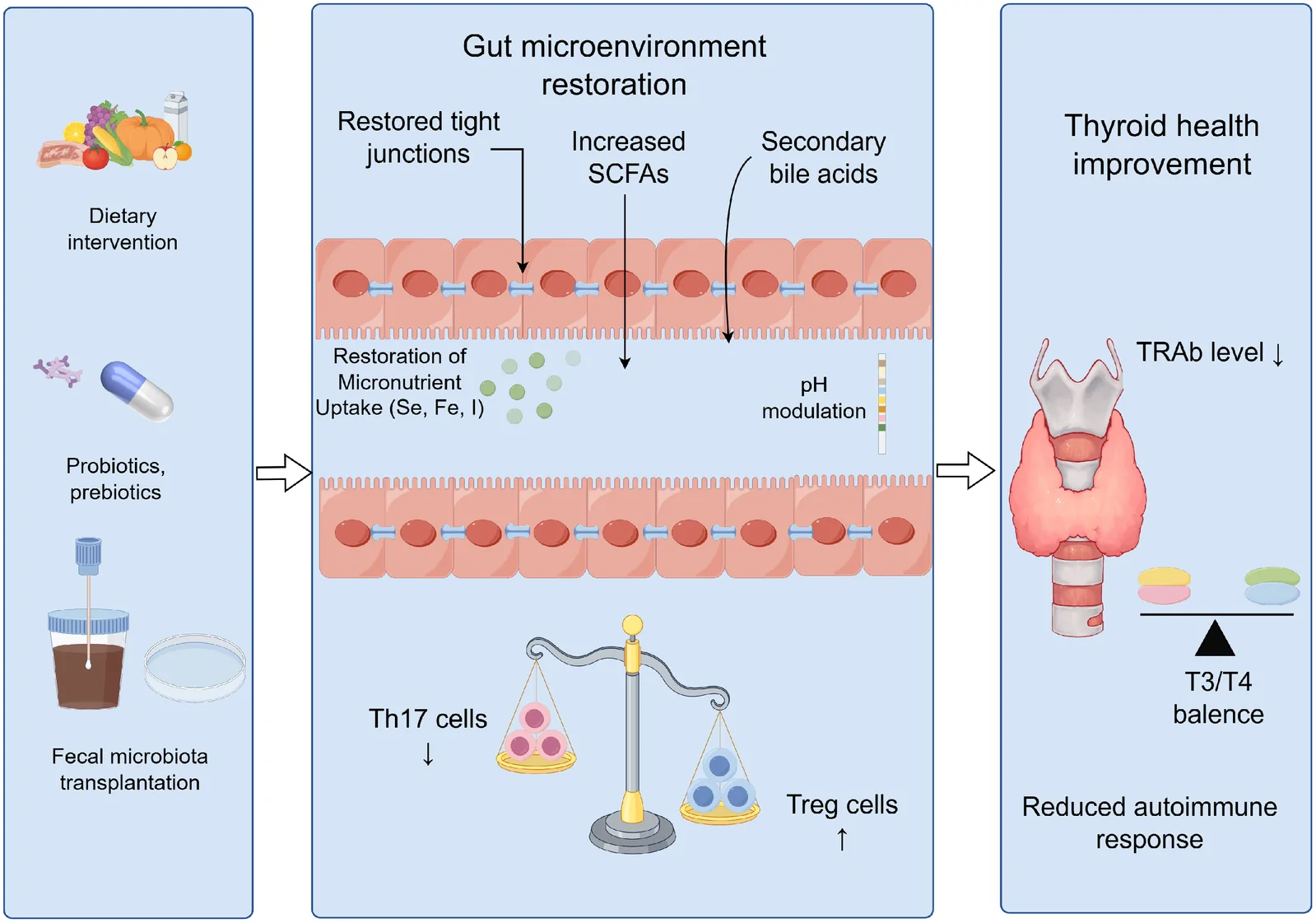
Potential gut microbiota-targeted therapeutic strategies for Graves’ disease. Therapeutic strategies focusing on the gut-thyroid axis aim to restore microbial homeostasis and alleviate thyroid autoimmunity through various targeted modalities. Supplementation with probiotics, prebiotics, and synbiotics can modulate microbiota composition. Dietary interventions, part icular ly the Mediterranean diet, exert protective effects through plant-based fibers and phenolic compounds that bolster antioxidant pathways and rebalance the Treg/Th17 equilibrium. Fecal microbiota transplantation (FMT) provides a comprehensive approach to resetting the intestinal ecosystem and has been shown in experimental models to normalize thyroid hormone levels, restore immune-cell balance, and reduce GD incidence. Created by Figdraw.

The mainstay therapies for GD have remained relatively static over the past seven decades. The therapeutic efficacy of ATDs has been suboptimal, with only approximately 50% of patients achieving long-term remission, while radioiodine and surgical treatments often lead to permanent hypothyroidism (Chaker et al., 2024). Consequently, it is imperative to develop effective and targeted therapies capable of addressing the underlying etiology of GD (**Table 2** and **Figure 2**).

**Table 2:** Potential mechanisms and effects of gut microbiota-targeted interventions in Graves’ disease.

Intervention category	Mechanisms
Probiotics, Prebiotics, Synbiotics	• Microbiome Modulation: Increase the abundance of beneficial bacteria and suppress pathogens. • Immunomodulation: May reduce TRAb levels and pro-inflammatory cytokines. • Metabolic Regulation: Enhance the production of beneficial metabolites like short-chain fatty acids (SCFAs); has been associated with modulation of bile acid metabolism and iron-related metabolic changes; optimize the intestinal physicochemical environment to favor probiotic colonization. • Intestinal Barrier Enhancement: May improve intestinal barrier integrity by inhibiting the TLR4/NF-κB pathway, thereby mitigating “leaky gut”.
Dietary Interventions	• Microbiome Remodeling • Immune and Inflammatory Regulation: Modulate the Treg/Th17 balance and reduce pro-inflammatory factors. • Antioxidant Protection: Phenolic compounds enhance antioxidant pathways, protecting the thyroid from oxidative stress.
Fecal Microbiota Transplantation	• Ecosystem Reset: Transfers the entire microbial community from a healthy donor to a recipient, comprehensively reshaping the microbiota structure. • Immune Status Restoration: Has been associated with partial restoration of Th17/Treg balance in experimental models. • Endocrine and Metabolic Normalization: Downregulates serum thyroid hormone levels and enhances hepatic thyroid hormone metabolism in animal models.

### 6.1 Probiotics, prebiotics, and synbiotics

Probiotics are categorized into two primary classes based on their origin and characteristics: traditional probiotics and next-generation probiotics (NGPs). NGPs encompass a more diverse range of microbial species, including members of the phyla Bacteroidetes and Actinobacteria, as well as specific genetically modified variants engineered for enhanced functionality (Fentie et al., 2024). Prebiotics are essential for maintaining microbial homeostasis by selectively stimulating the growth and metabolic activity of beneficial microbiota. Their scope has expanded from conventional substrates—such as human milk oligosaccharides (HMOs), synthetic oligosaccharides, conjugated linoleic acid, polyunsaturated fatty acids, and polyphenolic compounds—to include traditional Chinese medicine (TCM)-derived polysaccharides and alkaloids with potent immunomodulatory activities. The targeted application of prebiotics can effectively modulate gut microbiota composition, augment metabolic activity, and increase the production of SCFAs. Furthermore, synbiotics, which represent synergistic formulations of probiotics and prebiotics, enhance SCFA yields through complementary mechanisms (Shi et al., 2024).

Patients with GD are susceptible to intestinal malabsorption and gastrointestinal disturbances, including celiac disease, constipation, and abdominal distension, all of which may be significantly improved through probiotic supplementation (Knezevic et al., 2020). Clinical evidence indicates that a 6-month combined regimen of *Bifidobacterium* species and methimazole not only significantly restored thyroid function but also reduced TRAb levels. The same study also found that thyroid recovery was negatively correlated with fluctuations in serum iron and bile acid concentrations (Huo et al., 2021). Traditional Chinese medicine (TCM)-derived polysaccharides serve as a unique class of natural prebiotics characterized by a “dose-structure-microbiota” dependency. The degradation efficiency varies among microbial taxa; specifically, low-molecular-weight polysaccharides typically exhibit higher fermentation rates, whereas complex glycosidic linkages may impede microbial degradation (Rong et al., 2024). Berberine has emerged as a promising prebiotic compound. Compared to MMI monotherapy, the combination of berberine and MMI offers three distinct therapeutic advantages: (i) promoting the proliferation of beneficial commensal *Lactococcus* species; (ii) reducing the abundance of pathogenic species such as *Enterobacter hormaechei* and *Chryseobacterium indologenes*; and (iii) achieving superior outcomes in thyroid function markers, particularly TRAb levels (Han et al., 2022). Furthermore, polysaccharides derived from *Astragalus membranaceus*, *Atractylodes macrocephala*, and *Lycium barbarum* can selectively enrich probiotics like *Bifidobacterium* while inhibiting the activation of the TLR4/NF-κB pathway. These components effectively repair damaged intestinal villi and enhance barrier integrity, thereby mitigating the ‘leaky gut’ state and intercepting systemic inflammation at its source. These regulatory effects are primarily mediated through the remodeling of microbial metabolic pathways, including the promotion of SCFA synthesis and the optimization of bile acid conversion and amino acid metabolism (Yue et al., 2022; Li et al., 2024; Zhang et al., 2024a). *Prunella vulgaris*, a staple in the clinical management of GD, contains polysaccharide fractions that create a favorable ecological niche for probiotic colonization by modifying the intestinal physicochemical environment, such as regulating pH levels (Zhu et al., 2025). In the future, the development of precision polysaccharide formulations based on patient-specific gut microbiota profiles may establish a novel paradigm for personalized microecological therapy in GD. Nevertheless, current clinical evidence for probiotics, prebiotics, and synbiotics in GD remains limited to small-scale studies and indirect mechanistic evidence; large, placebo-controlled trials are needed to confirm their efficacy and safety as adjunctive therapies.

### 6.2 Diet

Diet is a major determinant of gut microbiota composition, and diet-induced alterations in the gut microbiota can affect host metabolism, intestinal barrier integrity, and inflammatory responses (Jia et al., 2023). These processes are also disrupted in GD. A diet rich in plant-based foods increases the proportion of Firmicutes members that can metabolize fiber and complex polysaccharides, while an animal-based diet increases the relative abundance of Bacteroidetes and other bile-resistant symbionts (Fackelmann et al., 2025). Extreme dietary regimens, such as the ketogenic diet, may reduce the abundance of *Bifidobacterium* in the microbiome, modulate Th17-cell development and adversely affect intestinal barrier function (Ang et al., 2020; Linsalata et al., 2023). The Mediterranean diet has been shown to help protect the thyroid gland against oxidative stress-induced damage (Lagana et al., 2025). The Mediterranean diet demonstrates significant microbiota-modulating effects, particularly through enrichment of beneficial taxa such as *Lactobacillus* spp., while concurrently enhancing intestinal barrier integrity. The omega-3 polyunsaturated fatty acids, which are abundant in fish, can rebalance the Treg/Th17 cell equilibrium and suppress NF-κB-mediated signaling, thereby attenuating proinflammatory cytokine production. The phenolic compounds in olive oil (such as hydroxytyrosol and oleuropein) may enhance antioxidant pathways and exert potential protective effects on thyroid autoimmunity (Ruggeri et al., 2023). The effects of a gluten-free diet on thyroid health remain contentious. A recent clinical investigation demonstrated no significant modulation of oxidative stress markers or thyroid autoimmunity parameters following this dietary intervention (Lagana et al., 2025). In contrast, a meta-analysis revealed potential benefits for both thyroid function and associated inflammatory responses (Piticchio et al., 2023). Overall, dietary modulation is biologically plausible but remains supported mainly by observational and indirect interventional evidence; GD-specific randomized dietary trials are still needed before firm clinical recommendations can be made.

### 6.3 Fecal microbiota transplantation

Fecal microbiota transplantation (FMT) involves the transfer of processed intestinal microbial communities from healthy donors to recipients with the aim of restoring disrupted gut microbial homeostasis. At present, FMT is the principal “whole-gut microbiome replacement” strategy and has been incorporated into clinical practice guidelines for recurrent *Clostridioides difficile* infection (Yadegar et al., 2024). Proposed mechanisms include bacterial engraftment and functional restoration of gut homeostasis, recovery of colonization resistance, modulation of microbial metabolites, and immunoregulatory effects mediated through host–microbiota interactions (Liu et al., 2023). In L-thyroxine-treated hyperthyroid Mongolian gerbils, cecal microbiota transplantation from control donors increased hepatic type 2 deiodinase expression, accelerated the decline of serum T3 and T4 levels, and promoted recovery of resting metabolic rate and body temperature toward normal levels (Khakisahneh et al., 2022). In a GD mouse model, transplantation of microbiota from healthy donors, compared with microbiota from GD patients, reduced serum thyroxine, TRAb, and IL-17A levels, increased IL-10 concentrations, and lowered GD incidence, indicating that restoration of a healthier microbial ecosystem may partially ameliorate thyroid dysfunction and immune-inflammatory dysregulation (Su et al., 2020). These findings suggest that FMT may have potential as an adjunctive microbiota-based strategy for GD; however, current evidence is limited to preclinical animal models, and its efficacy, safety, donor selection, and optimal delivery protocols have not yet been established in patients with GD and require validation in rigorously designed clinical studies.

## 7 Limitations

Several limitations of the current evidence should be acknowledged. First, as this article is a narrative review rather than a systematic review or meta-analysis, formal risk-of-bias assessment and quantitative evidence synthesis were not performed, and potential selection bias cannot be excluded. Second, most human microbiome studies in GD or GO are cross-sectional and observational, which limits causal inference and makes it difficult to determine whether microbial alterations precede disease onset or arise secondary to thyroid dysfunction, treatment, or lifestyle changes. Third, the reported findings may be influenced by potential confounders, including diet, geographic region, smoking, iodine and micronutrient status, antibiotics, and antithyroid medications. Many available studies also involve relatively small cohorts and differ in sampling procedures, sequencing platforms, taxonomic resolution, and bioinformatic pipelines, thereby limiting comparability and reproducibility. In addition, although animal models are useful for hypothesis testing, they do not fully recapitulate human GD, GO, or the complexity of the human gut ecosystem. Finally, microbiota-directed interventions in GD remain at an early stage, and large, well-controlled clinical trials are still lacking.

## 8 Conclusions and future perspectives

In summary, accumulating evidence suggests that gut microbial dysbiosis is associated with GD and may interact with thyroid autoimmunity through microbial metabolites, intestinal barrier dysfunction, immune disequilibrium, molecular mimicry, and altered micronutrient handling. However, current evidence supports association rather than causation, and the clinical utility of microbiota-based biomarkers or interventions remains to be established. Future research should focus on several testable priorities. First, longitudinal prospective cohorts should serially collect fecal samples, oral samples, metabolomic data, and immune profiles from newly diagnosed patients, during antithyroid drug treatment, and after remission or relapse; where feasible, studies in high-risk individuals before disease onset would be particularly valuable. Such designs would help clarify the temporal relationship between microbial changes and GD and identify microbial signatures predictive of disease activity, TRAb persistence, treatment response, or recurrence. Second, randomized placebo-controlled trials are needed to evaluate specific probiotics, prebiotics, synbiotics, dietary strategies, or other microbiota-targeted adjunctive therapies in combination with conventional antithyroid drugs, with thyroid function, TRAb levels, relapse risk, safety, and microbiota/metabolite changes as prespecified outcomes. Third, mechanistic studies using germ-free or gnotobiotic mouse models colonized with human GD-associated microbiota could help test causal pathways linking microbial dysbiosis, barrier dysfunction, Th17/Treg imbalance, and thyroid autoimmunity. Finally, multi-kingdom metagenomic and multi-omics approaches integrating bacterial, fungal, viral, and archaeal profiles with microbial metabolites and host immune phenotypes may identify conserved functional signatures and clarify the diagnostic or therapeutic relevance of the oral–gut microbial axis in GD.
